# A Multi-Level Systems Biology Analysis of Aldrin’s Metabolic Effects on Prostate Cancer Cells

**DOI:** 10.3390/proteomes11020011

**Published:** 2023-03-23

**Authors:** Carmen Bedia, Nuria Dalmau, Lars K. Nielsen, Romà Tauler, Igor Marín de Mas

**Affiliations:** 1 Department of Environmental Chemistry, Institute of Environmental Assessment and Water Research (IDAEA-CSIC), 08034 Barcelona, Spain; 2 The Novo Nordisk Foundation Center for Biosustainability, Technical University of Denmark, 2800 Lyngby, Denmark; 3 CAG Center for Endotheliomics, Copenhagen University Hospital, 2100 Rigshospitalet, Denmark

**Keywords:** prostate cancer, endocrine disruptor, genome-scale metabolic modeling, data-driven analysis, metabolic reprogramming

## Abstract

Although numerous studies support a dose–effect relationship between Endocrine disruptors (EDs) and the progression and malignancy of tumors, the impact of a chronic exposure to non-lethal concentrations of EDs in cancer remains unknown. More specifically, a number of studies have reported the impact of Aldrin on a variety of cancer types, including prostate cancer. In previous studies, we demonstrated the induction of the malignant phenotype in DU145 prostate cancer (PCa) cells after a chronic exposure to Aldrin (an ED). Proteins are pivotal in the regulation and control of a variety of cellular processes. However, the mechanisms responsible for the impact of ED on PCa and the role of proteins in this process are not yet well understood. Here, two complementary computational approaches have been employed to investigate the molecular processes underlying the acquisition of malignancy in prostate cancer. First, the metabolic reprogramming associated with the chronic exposure to Aldrin in DU145 cells was studied by integrating transcriptomics and metabolomics via constraint-based metabolic modeling. Second, gene set enrichment analysis was applied to determine (i) altered regulatory pathways and (ii) the correlation between changes in the transcriptomic profile of Aldrin-exposed cells and tumor progression in various types of cancer. Experimental validation confirmed predictions revealing a disruption in metabolic and regulatory pathways. This alteration results in the modification of protein levels crucial in regulating triacylglyceride/cholesterol, linked to the malignant phenotype observed in Aldrin-exposed cells.

## 1. Introduction

Prostate cancer (PCa) is the most common type of cancer and a second leading cause of cancer deaths in the male population. Almost one million new cases are diagnosed globally per year, imposing a high burden on healthcare systems worldwide [1,2].

Similarly to numerous other types of cancer, prostate cancer has also been linked to particular environmental exposures. It is widely accepted that environmental factors play a significant role in tumor initiation, progression, and phenotype. In this sense, a number of studies describe a dose–effect relationship between endocrine disruptors (EDs) and the onset and progression of prostate cancer (PCa). These compounds can disrupt the endocrine system by mimicking the effect of certain hormones which alters the cancer metabolism, enhancing tumor progression and malignancy [3,4]. More specifically, several studies have found a correlation between an exposure to Aldrin, an endocrine disruptor, and an increased risk of aggressive prostate cancer [5]. Although sub-lethal concentrations of endocrine disruptors (EDs) have been linked to prostate cancer (PCa) progression and malignancy acquisition, our current knowledge on the underlying mechanisms remains limited. In a previous study, we demonstrated that a prolonged exposure to a non-lethal concentration of Aldrin can lead to significant alterations in the lipidomic profile of DU145, a prostate cancer cell line. Additionally, it was found that this exposure also enhanced the malignant phenotype of these cells, highlighting the potential carcinogenic effects of this chemical [6]. Aldrin is an organic pollutant that exhibits ED characteristics. It disrupts gene regulation, altering protein levels and activity through processes such as phosphorylation, ultimately leading to an abnormal tumor metabolism (metabolic reprogramming) [7,8].

Thus, to fully understand the metabolic processes involved in a complex and multifaceted disease such as cancer, it is crucial to take a global perspective. This requires an integrated approach that considers the entire metabolic network, as well as the cross-talk with regulatory mechanisms.

In this context, genome-scale metabolic models (GEMs) have emerged as a potential solution to decipher the complexity of the molecular mechanisms underlying cancer within the framework of systems biology [9,10]. This computational tool represents the biochemical, genetic, and genomic knowledge of a given organism [11,12,13]. GEMs present a highly beneficial framework for integrating omics data [14], and they serve as an excellent analytical tool for studying metabolic fluxes within a network through the application of constraint-based techniques such as flux balance analysis (FBA) [15]. This model-driven approach has proven to be effective at uncovering valuable insights into the molecular mechanisms driving the aberrant cancer metabolism and identifying potential vulnerabilities [16,17,18]. However, the alterations in the metabolic secondary response associated with a chronic exposure to a sub-lethal concentration of pollutants such as EDs have not yet been taken into account in these computational approaches.

While GEMs allow for a comprehensive understanding of the metabolic network’s activity in a given condition (i.e., Aldrin-exposed or non-exposed conditions), they are not suitable to study the dysregulated gene regulatory and signaling pathways that contribute to the abnormal tumoral metabolism, and thus lack the ability to provide a more comprehensive understanding of the molecular mechanisms involved. In this context, gene set enrichment analysis (GSEA) [19] proves to be a more suitable approach to integrate the entire gene expression profile and identify the underlying cellular mechanisms, including the gene regulations and signaling pathways, that are associated with a specific phenotype.

This study represents a first approach in the integration of multi-omics data, leveraging two powerful and complementary systems biology methods, GEMs and GSEA, to unveil the molecular mechanisms underlying the gain of malignancy in PCa due to the chronic exposure to non-lethal concentrations of Aldrin, using the well-established DU145 PCa cell line as a cellular model.

**Figure 1:**
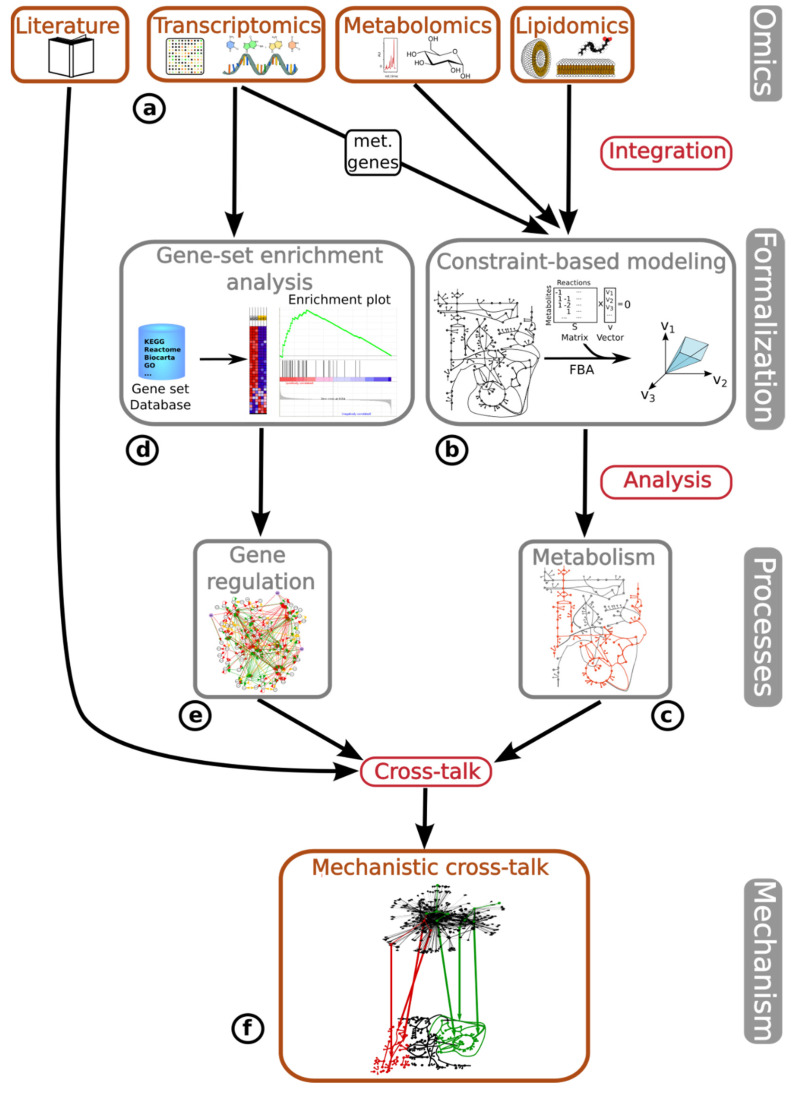
Overall process of omics data integration and analysis. (**a**) Omics data integrated in this study: literature-based, transcriptomics, metabolomics and lipidomics. (**b**) Metabolomics, lipidomics, and transcriptomics (only metabolic genes) are integrated into a GEM of human metabolism by applying CBM methods. (**c**) Result of GEM-based CBM: activity state of the metabolic network in Aldrin-exposed and non-exposed conditions. (**d**) The full transcriptomic dataset is integrated into a gene set enrichment analysis. (**e**) Result of GSEA: regulatory mechanisms associated with the gain of malignancy due to the chronic exposure to Aldrin in DU145 PCa cells. (**f**) Cross-talk: combine the output from the GSEA and CBM analyses by means of bibliographic search to find the cross-talk between the regulatory and metabolic layers that define a unique mechanism involving both layers that underlies the increase in malignancy reported in DU145 PCa cells due to the chronic exposure to non-lethal concentrations of Aldrin.

Here, we have integrated metabolomic, lipidomic, transcriptomic and literature-based data (Figure 1a) by applying two different approaches: (i) integrating the gene expression of metabolic genes and lipidomic and metabolomic data via constraint-based metabolic modeling methods to fully characterize the activity state of the metabolic network, and (ii) applying GSEA to identify potential changes in the regulatory pathways and correlate transcriptomic profiles between DU145 Aldrin-exposed cells and tumor progression in various tumor types obtained from the Cancer Genome Atlas (TCGA) [20]. First, the metabolomic, lipidomic, and transcriptomic data, obtained from metabolic genes, are integrated into one of the most widely utilized reconstructions of the human metabolism [13]. This is done through the utilization of established algorithms specifically designed for this purpose [21,22,23] (Figure 1b). Our integrative approach was employed to simulate, analyze, and predict metabolic fluxes using flux balance analysis (FBA) for metabolic phenotype predictions. We identified changes in the metabolic flux profile of DU145 PCa cells following a long-term exposure to non-lethal concentrations of Aldrin (Figure 1c).

Second, the entire gene expression dataset of Aldrin-exposed and non-exposed cells was compared with the curated gene signatures associated with different cellular mechanisms (Figure 1d). As result, the gene regulatory and signaling mechanisms associated with the chronic exposure to Aldrin were uncovered (Figure 1e). Finally, the study utilized literature-based research to gather information regarding the metabolic changes predicted by the model-driven analysis and the gene regulatory alterations identified by GSEA, combining them into a unified mechanism that explains the increased malignant phenotype associated with the chronic exposure to Aldrin in DU145 PCa cells (Figure 1f).

The multi-level computational analysis unveiled the molecular mechanisms involving both metabolic and gene regulatory processes, including the dysregulation of *HMGCoA*, that are consistent with the previously reported enhanced malignant phenotype associated with the chronic exposure to Aldrin in prostate cancer cells. This method presents a high potential as it allows for the study of the crucial role that proteins play in human diseases through the integration and analysis of various omics data.

The proposed approach offers a comprehensive understanding of intricate and multifaceted diseases, as demonstrated by the current study. It has the potential to be extrapolated to other human diseases in both clinical and environmental settings.

## 2. Materials and Methods

### 2.1. Cell Experiments, Transcriptomic Analysis, and Metabolomic Data Preparation

DU145 prostate cancer cells were obtained from the American Type Culture Collection. The Aldrin, cell culture media, and reagents were obtained from Merk (Kenilworth, NJ, USA). DU145 cells were cultured in an RPMI 1640 medium supplemented with 10% heat-inactivated fetal bovine serum, 100 μg/mL of streptomycin, and 100 U/mL of penicillin. The cell culture was grown in an incubator humidified with 5% of CO_2_ at 37 °C.

The DU145 PCa cells were exposed to sub-lethal concentrations of Aldrin (not affecting cell growth) for 50 days (Aldrin-exposed), while the control condition was carried out for the same period in Aldrin-free media (non-exposed), as described in Bedia et al.’s 2015 study [6]. After 50 days of treatment, samples of both Aldrin-exposed and non-exposed cells were collected at two time points within the exponential growth phase: at 0 and 5 h.

Transcriptomics: for the transcriptomic analysis, the Aldrin-exposed and non-exposed DU145 cells were harvested at 85% confluence using a rubber scraper into 2 mL of ice-cold PBS. The cells were centrifuged at 1300 rpm for 3 min at 4 °C and were washed twice with cold PBS. The NucleoSpin RNA kit (Macherey-Nagel, Düren, Germany) was used to extract the total RNA [6]. The Agilent 2100 Bioanalyzer platform (Agilent Technologies, Santa Clara, CA, USA) was used to determine the RNA quality. RNA (2 μg) was retrotranscribed to cDNA using the Transcriptor First Strand Synthesis Kit (Roche) and stored at −20 °C [6]. Finally, gene expression profiles from Aldrin-exposed and non-exposed cells were obtained by using an HG-U219 array plate (Affymetrix Inc., Santa Clara, CA, USA) and cDNA samples were obtained from the experiment after 50 days of exposure. Microarray data were normalized using the RMA method [24] (GSE132063).

Metabolomics: for metabolomics, cells and culture media were used for the analysis. The cell media was collected in 1.5 mL tubes, centrifuged, and the supernatants were lyophilized. The cells for lipidomics and metabolomics were harvested using a rubber scraper into 2 mL of ice-cold PBS. Next, the cells were centrifuged for 3 min at 1300 rpm and 4 °C and were washed twice with cold PBS.

For metabolite extraction, 1 mL of 90% chloroform/methanol mixture 1:9 in water was added to the cell pellet or media dried samples. This mixture was fortified with 500 pmol ^13^C D-Glucose standard (CLM-420-PK, Cambridge Isotope Laboratories). The mixture was vortexed vigorously, sonicated for 5 min, and centrifuged for 10 min at 15,000 rpm. The supernatant was transferred to another tube and solvent was evaporated under N_2_ stream. Next, the samples were resuspended in 150 μL of methanol, centrifugated again 10 min at 15,000 rpm, and 100 μL was transferred to glass vials for injection. For quantitative purposes, homemade standard mix solutions of metabolites (including amino acid and nucleoside commercial mixtures of Sigma and the selection of metabolites introduced in the model) at different concentrations ranging from 2.5 to 20 ppm were prepared in methanol.

Chromatographic separations were carried on an the Accela UHPLC system (Thermo Scientific, Waltham, MA, USA) using a hydrophilic interaction liquid chromatography (HILIC) column (TSK Gel Amide-80 column: 250 × 2.1 mm, 5 μm) from Tosoh Bioscience (Tokyo, Japan) at room temperature. Two solvents were used to perform the elution gradient: acetonitrile (A) and 5 mM of ammonium acetate adjusted to pH 5.5 with acetic acid (B). Solvents A and B were mixed as follows: 0–8 min, linear gradient from 25 to 30% B; 8–10 min, from 30 to 60% B; 10–12 min, 60% B; 12–14 min, back from 60% to 25% B; and from 14 to 20 min, 25% B. The mobile phase flow rate was set to 0.15 mL·min^−1^ and the injection volume was 5 μL. The Exactive Orbitrap mass spectrometer (Thermo Fisher Scientific, Waltham, MA, USA) with heated electrospray (HESI) as an ionization source was used. HESI was used separately in positive and negative mode and metabolites were fragmented in HCD collision cell by alternating the MS scans of the precursor ions and all ion fragmentation (AIF) scans. Mass spectra acquisition was conducted in profile mode at a resolution of 50,000 full width half maximum (FWHM) at *m*/*z* 200. The following parameters were used: a sheath gas flow rate of 45 arbitrary units (a.u.); an electrospray voltage of 3.0 kV; an auxiliary gas flow rate of 10 a.u.; and a heated capillary temperature of 300 °C. The range of the full scan mass range was set between *m*/*z* 80 and *m*/*z* 1000. A normalized collision energy (NCE) of 25 eV was used to perform the AIF. Raw data were exported to a cdf format using the Excalibur file converter tool (Thermo Fisher Scientific, Waltham, MA, USA).

The cdf files from metabolomics and lipidomics were subjected to the ROI procedure [25] to extract the *m*/*z* features detected in each chromatographic run. Data were normalized by the number of cells and the amount of internal standard in each sample. Only the lipids and metabolites present in the GEM and that could be detected under the conducted metabolomics/lipidomics analytical procedure were quantified using the appropriate calibration curves. The annotation of metabolites and lipids was performed by exact mass matching in Human Metabolome Database [26] and LipidMaps [27] online databases, the MS2 fragmentation patterns reported in spectral libraries, and the information regarding retention times under the same chromatographic conditions collected in in-house databases from previous works. Further details about the analytical methodologies used for the metabolomics and lipidomics used in this work are provided in [28,29,30]. The resulting data were integrated into the GEM reconstruction analysis of Aldrin-exposed and non-exposed PCa cells.

### 2.2. Metabolic Model Readjustments/Refinement

In this study, we have used one of the most widely used reconstructions of the human metabolism [13] as a platform to integrate the metabolomic, lipidomic, and transcriptomic data. The GEM (Recon 2.2) accounts for 1789 enzyme-encoding genes, 7440 reactions, and 5063 metabolites distributed over 8 cellular compartments. In order to improve the omics data integration and analysis, a further adaptation of the model was required. More specifically, the lipid-associated metabolism was expanded, and reactions that did not display a steady-state flux other than zero (blocked reactions) were eliminated. These steps are detailed below.

Enabling GEM for lipidomic data integration: the intracellular lipidomic profiles of Aldrin-exposed and non-exposed DU145 cells were measured at two time points within the exponential growth phase (at 0 and 5 h). Since these measurements correspond to the whole-cell extracts/lysates, the contribution of each cellular compartment defined in the model is unknown. Thus, in order to integrate the lipidomic data into our computational analysis, the metabolic model was expanded as follows: (i) first an additional boundary compartment was defined, along with the corresponding boundary metabolites that encompass all the measured lipids present in multiple intracellular compartments (including cytosolic, mitochondrial, lysosomal, etc.); (ii) subsequently, the transport reactions between intracellular lipids and their corresponding boundary lipids were established. (iii) In addition, a sink reaction was introduced for each boundary lipid and, (iv) finally, a sink reaction was defined for the measured lipids that are restricted to a single intra-cellular compartment. This expanded GEM includes a boundary compartment accounting for all the measured lipids annotated in the metabolic model. Both exchange reactions associated with boundary lipids and sink reactions enabled the integration of the lipidomic data in the model-driven analysis in the form of constraints.

Model reduction: in order to reduce the computational time necessary to perform the analysis, the metabolic model (Recon2.2) [13] was reduced. This was achieved by removing the blocked reactions in the model. These reactions are those incapable of carrying any metabolic flux [31]. To this aim, a flux variability analysis (FVA) [32,33,34] was implemented using the ‘fluxVariability’ function in the COBRA toolbox [22,23]. This analysis computes the spectrum of fluxes that each reaction can carry while the value of the objective function is optimal. As a result, reactions that lack a solution different to zero are classified as blocked reactions [31] and are subsequently eliminated from the model. Finally, any metabolite that did not serve as a product or substrate in any reaction was removed from the model. These were identified as “dead-end metabolites”.

### 2.3. Characterize the Metabolic Flux Profile of Aldrin-Exposed and Non-Exposed Cells by Applying Flux Balance Analysis

Before the metabolomics network reconstruction can be used to compute its properties, an important step must be taken in which the network reconstruction is mathematically represented. This conversion translates the reconstructed network into a chemically accurate mathematical format, the stoichiometric S matrix that becomes the basis for the genome-scale model. In the S matrix, the reactions are in the columns and the metabolites in the rows. Each metabolite’s entry has its stoichiometric coefficient in the corresponding reaction. The stoichiometric matrix describes the quantitative relations between metabolites through the metabolic reactions and the steady-state assumption imposes flux balance constraints on the network, ensuring that the overall amount of any compound being produced is equal to the overall amount being consumed. The next step in FBA analysis is to define the objective function, usually the biomass production. The biomass production represents the rate at which metabolic compounds are converted into biomass constituents such as nucleic acids, proteins, and lipids. The objective of biomass production is mathematically represented by a global biomass reaction that becomes an extra column of coefficients in the stoichiometric matrix.

Once imposed on a network reconstruction, these balances and bounds define a space of allowable flux distributions in a network describing the possible rates at which every metabolite can be produced/consumed in the network. Here, the data integration plays an important part, in which FBA is able to perform simulations under different conditions by altering the constraints of the model.

### 2.4. Enhancing GEMs Predictive Capabilities via Omic Data Integration

The integration of biological information from different “omics” into a metabolic reconstruction analysis allows to further constraint the space of feasible solutions and provides a metabolic flux profile specific to a particular event, condition, or environment. GEMs encompass all known metabolic reactions that are encoded by an organism’s genome. Therefore, they serve as an ideal platform for integrating a diverse range of omics data, including transcriptomic and metabolic data. In this work, transcriptomic metabolomic and lipidomic data have been integrated in a metabolic network reconstruction analysis as follows.

Transcriptomic data integration into GEMs: gene expression profiles can be mapped onto a GEM via gene–protein–reaction associations (GPR). These associations enable each reaction to be connected to single or multiple genes associated with proteins/enzymes linked to a reaction. Gene expression levels can be utilized to infer metabolic reaction activity states via GPRs. This enables the development of a metabolic flux profile that accurately defines the phenotype associated with a specific gene expression profile.

The transcriptomic data were integrated by applying the iMAT algorithm, which incorporates gene expression data into a GEM via GPRs to predict global metabolic flux activity [21]. This approach aims to maximize the similarity between gene expression and the activity state of the metabolic network. The genes are set to be highly or lowly expressed by imposing an upper and lower threshold (i.e., 66th and 33rd percentiles as the upper and lower threshold, respectively). Thus, those genes with an expression level above the upper threshold are considered highly expressed, the genes below the lower threshold are considered lowly expressed, and the genes between these thresholds were considered moderately expressed. Each enzymatic reaction in the model was assigned a gene-expression-based value by propagating the gene expression through the gene–protein–reaction relationships (GPRs). Based on the reaction values, two sets of reactions were generated that consist of highly R_H_- and lowly R_L_-expressed reactions.

This information was used to formulate a mixed integer linear problem (MILP) [35], which is explained in more detail below:

max(∑*i*ϵ*R_H_* (*y_i_^+^* + *y_i_*^−^) + ∑*i*ϵ*R_L_* ^(*xi*)^) (1)

*S*⋯*v* = 0 (2)

*v_min_* ≤ *v* ≤ *v_max_* (3)

*v_i_* + *y_i_^+^*(*v_min,i_* − *ε*) ≥ *v_min,i_*; *i* ϵ *R_H_* (4)

*v_i_* + *y_i_^−^*(*v_max,i_* + *ε*) ≥ *v_max,i_*; *i* ϵ *R_H_* (5)

(1 − *x_i_*)*v_min,i_* ≤ *v_i_* ≤ (1 − *x_i_*)*v_max,i_*; *i* ϵ *R_L_* (6)

In Equation (1), a MILP is represented which maximizes the number of reactions whose activity is consistent with their expression state. The mass balance constraint is enforced in Equation (2), where *v* is the flux vector and *S* is a stoichiometric matrix. In Equation (3), maximum and minimum allowable flux is defined for each reaction by setting lower and upper flux bounds (*v_min_* and *v_max_*, respectively). For each highly expressed reaction, the Boolean variables *y^+^* and *y^−^* represent whether the reaction is active (in either direction, thus either *y^+^* or *y^−^* is assigned the value 1) or inactive (when both *y^+^* and *y^−^* are equal to 0). For each lowly expressed reaction, a Boolean variable *x* representing whether a reaction is inactive or active (1 or 0, respectively) is defined. A reaction associated with a highly expressed gene reaction is considered to be active if its predicted metabolic flux above or below the positive (*ε*) or negative (−*ε*) thresholds, respectively (Equations (4) and (5), respectively). A lowly expressed reaction is considered inactive if it does not carry a flux that is greater than 0 in either direction (Equation (6)). The optimization maximizes the number of reactions that exhibit similar levels of activity as their expression state.

This approach considers the mRNA levels as clues for the likelihood that the enzyme in question carries a metabolic flux in its associated reaction(s), and then solves a constraint-based modeling optimization problem (in this case a MILP) to find a steady-state metabolic flux distribution by assigning permissible flux ranges to all the reactions in the network consistent with their expression state. As a final result, the iMAT algorithm provides a steady-state flux profile that maximizes the number of reactions associated with highly expressed genes and the number of inactive reactions associated with lowly expressed genes, while the thermodynamic and stoichiometric constraints embedded into the model are satisfied.

Metabolomic and lipidomic data integration: endo- and exometabolomics and lipidomic sampling and measurements were conducted after 50 days of exposure and once again after 5 h. Thus, the metabolomic/lipidomic dataset considers two time points with triplicate sample measurements (Supplementary Material S1), allowing to determine the increase/decrease in the quantity of measured species between time points [31]. The experimental omics data were used to calculate the lower and upper bounds represented in Formulas (7) and (8).

ub = (c5 + Sd5) − (c0 − Sd0)(7)

lb = (c5 − Sd5) − (c0 + Sd0)(8)

Here, ub and lb are the upper and lower boundaries, respectively, while c0 is the average concentration of a specie measured in triplicate samples at time zero and c5 the average concentration of the same species measured in triplicate samples after 5 h. Additionally, Sd0 is a standard deviation of triplicate data at time zero, while Sd5 is a standard deviation of triplicate data at 5 h.

The metabolomic and lipidomic data were integrated as an exchange reaction of the lower and upper bounds into our metabolic network reconstruction analysis by applying the COBRA toolbox function ‘changeRxnBounds’ [22,23].

### 2.5. Identify the Activity State of the Metabolic Network by Applying Sensitivity and Robustness Analysis

This study utilizes an optimization method to determine the optimal values of system variables that maximize a particular objective function while satisfying certain constraints. In this case, the variables are the computed metabolic fluxes, and the objective function of the iMat algorithm is to maximize the similarity between the activity state of the metabolic network and the gene expression profile. In other words, it aims to maximize the number of active reactions associated with highly expressed genes and the inactive reactions associated with lowly expressed genes. Although a single solution is provided, there are multiple optimal solutions that define alternative combinations of metabolic fluxes. To account for the non-uniqueness of solutions and identify those reactions that are unambiguously active or inactive across all the solutions, a sensitivity analysis was conducted. Furthermore, the algorithm requires a predetermined set of thresholds to specify genes that are highly and lowly expressed. This could potentially offer an arbitrary aspect to the analysis. Here, a robustness analysis was conducted by performing a sensitivity analysis using various thresholds. Only reactions that maintained consistent activity states across the different thresholds are considered. Below a more detailed explanation of the sensitivity and robustness analysis is provided:

Sensitivity analysis: in gene expression data integration analysis, an optimal solution can be achieved by maximizing the objective function. However, it should be noted that this optimal solution may not be unique, meaning there could be multiple solutions with the same objective function value. Thus, a further exploration is necessary to determine the most suitable solution for the specific needs of the analysis. This is achieved by applying a sensitivity analysis [21]. The process involves addressing two MILP problems per reaction (as described in the transcriptomic data integration into GEMs sub-section), wherein one enforces the reaction to be active and the other enforces the reaction to be inactive. The ultimate reaction state is determined by the simulation with the best objective function value. If both simulations (active and inactive reaction) have optimal results, then the reaction state remains undetermined. This analysis allows for inferring the activity state of each reaction and the pathways in the different groups.

Robustness analysis: the algorithm used to integrate the gene expression levels into GEMs uses upper and lower thresholds to define highly or lowly expressed genes, while genes with expression values falling within these are considered moderately expressed. It raises the necessity to perform a robustness analysis in order to demonstrate the lack of dependency of a model’s predictions to the thresholds used in the analysis. In order to determine the robustness of the model’s predictions, a sensitivity analysis using the following pairs of thresholds defining the upper and lower boundaries, respectively, was conducted: percentiles 30th and 70th, 33rd and 66th, and 40th and 60th. Thereby, a set of reactions unambiguously predicted to be either active or inactive across the different thresholds was defined.

A more detailed explanation of the overall methodology including the sensitivity, robustness, and significance analysis can be found in Marin de Mas et al. (2018) [31].

### 2.6. Unveiling Potential Gene Regulation and the Potential Effect on Metabolism by Analyzing Gene Expression Data via GSEA

Transcriptomic expression data for Aldrin-exposed and non-exposed DU145 cells (GSE132063) were analyzed using the list of curated gene signatures (Molecular Signatures Database V5.1). Enrichment in the gene set was considered significant for FDR values ≤ 0.1, NES > 1.5 and a leading edge signal > 50%.

Next, the resulting relevant gene sets were evaluated in order to identify the genes contributing to the significance of the gene set.

### 2.7. Identifying the Cross-Talk between Gene Regulatory and Metabolic Mechanisms by Means of Literature-Based Analysis

Finally, in order to establish a comprehensive understanding of the underlying mechanisms driving different metabolic activities between conditions, a literature-based analysis was conducted. This analysis sought to connect the relevant genes identified through the GSEA analysis with the metabolic reactions that exhibit different activity states between conditions, as determined by the model-driven analysis. This analysis provided a more holistic perspective on the functional implications of the observed differences.

### 2.8. Using GSEA to Identify Correlations between the Effects of the Chronic Exposure to Aldrin in DU145 PCa Cells and PCa Progression and Metastasis

A metabolic gene set with a significant differential expression between Aldrin-exposed and non-exposed DU145 cells was generated by applying the multivariate discriminant PLS-DA method [24,36,37]. Relevant genes were those having a VIP (variable’s importance score) [38] value equal or higher than 1.5. The resulting Aldrin-exposed-enriched metabolic gene set was applied on an expression dataset for tumor types (Supplementary Material S2) retrieved from the Cancer Genome Atlas [20] using GSEA. For this analysis, we specifically selected datasets that include clinical tumor stage information. The samples were then grouped based on their tumor stages—T1, T2, T3, or T4—and metastatic status. GSEA was performed on these datasets by applying a weighted scoring scheme and a Pearson metric with 1000 phenotype permutations. Enrichment along tumor progression of the Aldrin-exposed DU145 cells metabolic gene set in these datasets was considered significant for FDR values ≤ 0.250.

### 2.9. Effects of INSIG2 Inhibition on HMGCoA Levels and Cholesterol Metabolism

siRNA experiments: siRNA sequences (ON-TARGET plus SMART pool, 5 nmol) against INSIG2 (L-021039) and non-targeting sequences (D-001810) and the transfection reagent DharmaFECT1 were purchased from Dharmacon Thermo Scientific (Lafayette, CO, USA).

Western blot: cells were harvested by trypsinization, centrifuged, and washed twice with PBS 1X. Next, cell lysed was performed using mammalian lysis buffer 1X (ab179835, Abcam, Cambridge, UK) together with the cocktail protein inhibitor 1X (Thermo Scientific, Waltham, MA, USA). The proteins in cell lysates were quantified using the BCA assay (Thermo Scientific), and 50 mg of proteins per sample was resolved by 10% SDS-PAGE. Next, PVDF membranes (Roche, Basel, Switzerland) were used to transfer the proteins. Membranes were blocked with TBS 1X containing 0.1% Tween 20 and probed with the antibodies to INSIG2 (rabbit polyclonal antibody, 24766-I-AP, Proteintech, Rosemont, IL, USA), HMGCR (rabbit polyclonal antibody, ab174830, Abcam), and β-actin (ab8227, Abcam). Membranes were developed using the chemiluminescent signal detection kit ECL Prime Western Blotting detection reagent (GE Healthcare, Chicago, IL, USA) and visualized using a LICOR C-DiGit blot scanner. The relative quantification of the relative Western blot band intensity quantification was determined using the software Image Studio Lite version 5.0 (LI-COR Biosciences, Lincoln, NE, USA); values were normalized to those of β-actin, and differences between the samples were calculated.

## 3. Results

### 3.1. Omics Data Integration and Model Validation

Our study aimed to explore the metabolic mechanisms that contribute to the promotion of a malignant phenotype in DU145 prostate cancer cells following a chronic exposure to non-lethal concentrations of Aldrin. The multi-level approach utilized in this study underwent several phases, which are detailed upon further below.

First, the experimentally measured metabolomic, lipidomic, and transcriptomic data were pre-processed for integration into a Genome-scale metabolic reconstruction analysis. Next, a PLS-DA analysis revealed the relevant compounds that were able to discriminate between treated and non-treated samples. As a result, 167 metabolites and 82 lipids were selected and mapped into the GEM (Supplementary Material S1). The gene expression profiles from Aldrin-exposed and non-exposed cells were obtained by using a microarray platform followed by microarray data normalization using the RMA method [24]. Finally, the recon 2.2 GEM was readjusted/refined to integrate the above-mentioned pre-processed omics data. Metabolomics and lipidomics were integrated by i. first mapping the compounds on the GEM and adding exchange or sink reactions when required and ii. integrating the experimental measurements means ± SD, respectively, as upper and lower boundaries of the corresponding exchange reactions. Transcriptomic data were integrated through the gene–protein–reaction associations (GPRs) [36].

Next, by applying the iMat algorithm [21], the metabolic network activity state profile of Aldrin-exposed and non-exposed DU145 cells was inferred. The reliability of the model’s predictions was determined by performing a sensitivity and a robustness analysis (see Materials and Methods). Here, different sets of lower/upper thresholds were tested: percentiles 40–60, 33–66, and 30–70, respectively (Supplementary Material S3).

In order to validate the reliability of the model’s predictions, the consumption/secretion rates of 55 metabolites not integrated previously in the analysis were compared with the predicted values from the model, and Fisher’s test was used to determine the statistical significance of the results (see Supplementary Material S3). This analysis was performed on all the tested thresholds, showing that the computational model was able to predict correctly between 79 and 83% of the consumption/production rates with an associated *p* < 0.01, which further validates the reliability of the computational model’s predictions.

### 3.2. Metabolic Network Analysis Unveils Marked Differences in Lipid-Associated Pathways between Aldrin-Exposed and Non-Exposed DU145 PCa Cells

The comparative analysis of flux profiles of 7696 reactions in the model revealed the reactions with differential activity states between Aldrin-exposed and non-exposed conditions (Supplementary Material S3). Here, the differential activity state analysis determines those reactions that are active only in one of the groups (Aldrin-exposed or non-exposed). Next, the analysis was performed on the pathways represented as sub-systems in the GEM (Supplementary Material S3), as detailed by Marin de Mas et al. in 2018 [31]. The activity state of a pathway was defined as the number of active reactions divided by the total number of reactions in the pathway.

To assess how the thresholds impact the inference of the metabolic pathway’s activity state, we conducted Fisher’s exact test for each pathway across different threshold values and applied a corrected Bonferroni *p*-value [37] to analyze and evaluate the results obtained (see Supplementary Material S3). The statistical analysis showed no significant differences in the pathway activity predictions across the different thresholds, except for the D-alanine pathway. Thus, the mean metabolic activity ratio between Aldrin-exposed and non-exposed cells together with the maximum Bonferroni p-value were used to define those metabolic pathways with significant differences. Here, those pathways with differences higher than 10% and a corrected p-value lower than 0.05 were considered differentially activated.

**Figure 2:**
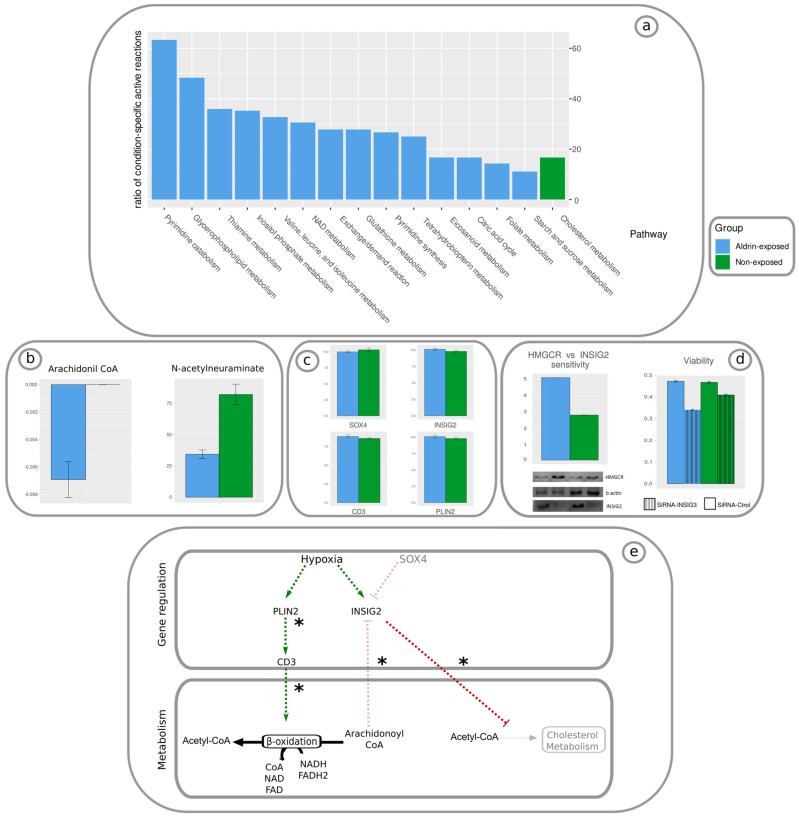
Summary of computational results, experimental validations, and proposed mechanism. (**a**) List of relevant pathways with significant differences between Aldrin-exposed (blue) and non-exposed (green) DU145 PCa cells. Bars represent the ratio of active reactions between conditions. Transport reactions have been excluded from this figure to avoid distortions due to the high differences between conditions (full figure can be found in Supplementary Material S3). (**b**) Experimental validation of relevant predicted metabolites with significant differences in consumption/secretion rates between conditions in AU. (**c**) Experimental validation of relevant genes with significant differences between conditions. (**d**) Experimental validation of HMGCR inhibition and cell viability measurements using INSIG2 siRNA. HMGCR vs. INSIG2 sensitivity: Aldrin-exposed cells are 1.8 times more sensitive to INSIG2 inhibition (according to the Western blot below the graph), viability: cell viability is less compromised in non-exposed cells. (**e**) Proposed mechanism: green arrows: activation, red arrow: inhibition, black arrows: metabolic reactions, light arrows: inactive/reduced activity, dark arrows: active/enhanced activity, *: mechanims based on literature search.

The computational analysis unveiled marked differences in the activity state of DU145 prostate cancer cells in response to the chronic exposure to Aldrin. Aldrin-exposed cells were predicted to have a more active metabolism with a higher number of active reactions. More specifically, transport reactions through the cell membrane or to peroxisome, Golgi apparatus, or endoplasmic reticulum were found to be more active in this group. Other relevant metabolic pathways that were predicted to be active in Aldrin-exposed cells were the metabolism of eicosanoids, glycerophospholipids, folate, N-glycan synthesis, NAD, and some amino acids such as valine, leucine, isoleucine, thiamine, and glutathione. On the other hand, the cholesterol metabolism was predicted to be enhanced in DU145 PCa non-exposed cells (Figure 2a). This finding is consistent with cholesterol levels in non-exposed PCa cells described by Bedia et al. in 2015. The cholesterol metabolism, as well as triacylglycerides (TAG) metabolism, is fueled by a common pool of Acetyl-CoA, which is consistent with previous evidence showing an inverse correlation of cholesterol and TAG levels in both Aldrin-exposed and non-exposed cells [6]. The reliability of the model’s predictions was determined by performing a sensitivity and a robustness analysis (see Methods).

A further analysis comparing the activity state of the metabolic reactions between Aldrin-exposed and non-exposed cells was performed. To identify consistent differences between the conditions, the metabolic reactions were analyzed for their activity states across various thresholds. This allowed for the identification of reactions that showed significant changes in activity levels between conditions and remained consistent across the different threshold values. From this analysis, two main reactions were found to be active in Aldrin-exposed cells and inactive in non-exposed cells. One of the reactions corresponds to the beta-oxidation of Arachidoyl-CoA into Acetyl-CoA (Figure 2b, Supplementary Material S3). This metabolite is predicted to not be produced endogenously, which is consistent with the higher arachidonic acid uptake in Aldrin-exposed cells compared with non-exposed cells, 0.007 mM/h ± 0.0013 and 0, respectively (Figure 2b, Supplementary Material S3). Another reaction that was predicted to be active only in Aldrin-exposed cells was the degradation of N-acetylneuraminate to N-acetyl-D-mannosamine and pyruvate. This prediction was further substantiated through experimental measurements indicating higher intracellular levels of N-acetylneuraminate in cells that did not undergo an exposure. (Figure 2b, Supplementary Material S3).

### 3.3. Identification of Regulatory Pathways Associated with the Chronic Exposure to Aldrin in DU145 PCa Cells by Applying GSEA on Transcriptomic Data

Previous analysis integrates both metabolomic and transcriptomic data. However, since the transcriptomic data are integrated through the gene–protein–reaction associations described in GEMs, only the expression levels of metabolic genes (genes encoding enzymes) can be used. In this sense, GSEA complements the GEM-based analysis by enabling the analysis of the full transcriptomic dataset. More specifically, GSEA was used to identify gene regulatory mechanisms and signaling pathways strongly correlated with the genes deferentially expressed between conditions [19]. To this aim, the Aldrin-exposed and non-exposed cells transcriptomic data (four replicates each) were compared with the curated gene sets from the Molecular Signatures Database V5.1. As a result, the GSEA model revealed that hypoxia activation and SOX4-mediated up-regulation mechanisms were significantly down-regulated in non-exposed cells compared with Aldrin-exposed cells (MENSE_HYPOXIA_UP and PRAMOONJAGO_SOX4_TARGETS_UP gene sets, respectively, Supplementary Material S2). These two mechanisms are highly overlapped: 14 common genes, while there were 42 and 23 enriched genes in MENSE_HYPOXIA_UP and PRAMOONJAGO_SOX4_TARGETS_UP gene sets, respectively, which indicates a common mechanism described in both gene sets. The common genes are described in Table 1.

### 3.4. Multilevel Approach Combining GSEA and GEMS Analyses Unveils Molecular Mechanisms Associated with the Chronic Exposure to Aldrin in DU145 PCa Cells

In order to unveil the molecular mechanism underlying the gain of malignancy in DU145 PCa cells associated with the chronic exposure to Aldrin, the results extracted from the GEM-based analysis and from the GSEA method were combined. These two complementary approaches describe two different processes occurring at two different layers within the cell: (i). metabolisms formalized as a genome-scale constraint-based metabolic network model, and (ii). gene regulation analyzed by applying GSEA. In order to study the cross-talk between the metabolism and gene regulation, literature-based data were also integrated in the analysis. This information was used to link the metabolic alterations predicted by the GEM-based analysis with the mechanisms and relevant genes defined in the GSEA method. Based on the analysis, it was discovered that the two genes, PLIN2 and INSIG2, play a significant role in regulating the metabolism. The research predicted an increase in the expression of these genes in cells exposed to Aldrin as opposed to those that were not exposed (Figure 2c). PLIN2 enhances arachidonoyl-CoA beta oxidation via CD3 [39,40] (Supplementary Material S3—“Reaction_Activity_State_Threshold_Summary”). In this case, CD3 was up-regulated in Aldrin-exposed cells compared with non-exposed cells, which supports this hypothesis. On the other hand, arachidonoyl-CoA inhibits INSIG2 [41]. However, the lower arachidonoyl-CoA levels predicted in Aldrin-exposed cells and validated experimentally reduce its inhibitory capability on INSIG2. Additionally, INSIG2 is predicted to be up-regulated in Aldrin-exposed cells compared with the non-exposed condition via SOX4 and hypoxia. These findings were supported by experimental measurements of the gene expression showing higher levels in Aldrin-exposed cells. Finally, INSIG2 inhibits HMGCoA protein by ubiquitination [42,43], which is a limiting reaction in the cholesterol metabolism. In order to validate this hypothesis, a HMGCoA Western blot was carried out in normal conditions and inhibiting INSIG2 with sh-RNA. This experimental validation demonstrated a higher sensitivity of cholesterol levels towards INSIG2 inhibition in Aldrin-exposed cells (59% more sensitive, Supplementary Material S4), which suggests a higher utilization of this pathway in non-exposed cells.

The rise in cholesterol levels was found to be more significant in cells exposed to Aldrin as compared to those that were not exposed when INSIG2 was inhibited. This observation suggests that the inhibition of HMGCoA through INSIG2 is stronger in Aldrin-exposed cells under basal conditions. In addition, the relative HMGCoA increment was higher in Aldrin-exposed cells than in non-exposed cells when INSIG2 was inhibited (sensitivity in A.U.: 5.12 and 2.79 in Aldrin-exposed and non-exposed cells, respectively). This also indicates a stronger inhibition of HMGCoA in Aldrin-exposed cells in basal conditions. Finally, the cell viability decreased less in non-exposed cells when INSIG2 was inhibited, showing that non-exposed SiRNA-INIG2 cells were closer to the basal conditions where the INSIG2 expression is lower than in the Aldrin-exposed group (Figure 2d, Supplementary Material S4). Therefore, a unified mechanism that involves metabolic and gene-regulatory alterations was identified, leading to changes in the lipidomic profile of DU149 PCa cells associated with the acquisition of malignancy upon a chronic exposure to Aldrin.

More specifically, PLIN2 and INSIG2 were predicted to be upregulated via hypoxia and the lack of SOX4-induced inhibition. PLIN2 enhances arachidonoyl-CoA degradation by activating beta oxidation, which in turn reduces the inhibitory effects of this compound on INSIG2. The up-regulation of INSIG2 increases HMGCoA protein ubiquitination. This is the limiting reaction in the cholesterol synthesis pathway which is consistent with measured lower cholesterol levels in Aldrin-exposed cells (Figure 2e). The inhibition of cholesterol synthesis increases the levels of TAG as these two processes compete for the same pool of Acetil-CoA, and this is also reported in DU-145 after a long exposure to non-lethal concentrations of Aldrin [6].

### 3.5. Tumor Progression and Metastasis in Several Tumor Types Correlate with the Altered Genes Associated with the Chronic Exposure to Aldrin in DU-145 Pca

To investigate whether the alterations observed in Aldrin-exposed cells at the transcriptomic level are linked to tumor progression and metastasis, we employed the GSEA methodology. First, we utilized the PLS-DA method to identify a set of differentially expressed genes between cells exposed to Aldrin and those that were not. Specifically, we selected genes with a VIP score of 1.5 or greater. Using this approach, we identified a set of 101 genes that exhibited significant changes in expression levels (Supplementary Material S1).

The gene set associated with the malignant phenotypic changes observed in Aldrin-exposed cells was significantly enriched, concomitant with eight types of human tumors. Thus, the transcriptomic signature of the malignant phenotype found in Aldrin-exposed cells correlates well with the malignant progression of eight other types of human tumors retrieved from the Cancer Genome Atlas (TCGA) database [20], including uveal melanoma, lymphoma and lung, bladder, kidney, rectum, and cervical carcinoma (Table 2, Supplementary Material S5).

## 4. Discussion

A holistic analysis of molecular alterations triggering complex and multi-factorial diseases such as cancer enables us to increase our understanding of how environmental perturbations affect tumor progression and malignancy acquisition. Exploring the overall processes that occur within a cell is essential when investigating the effects of a prolonged exposure to widespread environmental pollutants such as EDs at non-lethal concentrations.

Bedia et al. [6] reported in 2015 that a non-lethal exposure to various EDs led to an amplified malignant phenotype in DU145 PCa cells. Here, Aldrin was found to have the most substantial impact on these cells.

Developing a comprehensive understanding of the mechanisms involved in the acquisition of malignancy is crucial for the development of more effective therapeutic interventions. It is also important for improving our ability to assess and regulate environmental pollutants, such as specifically Aldrin and other EDs linked to cancer development.

Systems biology tools, such as genome-scale metabolic network modeling using constraint-based methods (GEM-based CBM) and gene set enrichment analysis (GSEA), provide a powerful means to integrate multiple layers of the hierarchical mechanistic structure that govern cellular processes. By capturing the complexity and dynamics of biological systems, these tools enable us to analyze them in a comprehensive and holistic manner.

Our study sought to delve into the metabolic mechanisms that contribute to the enhanced malignant phenotype resulting from a prolonged exposure to non-lethal concentrations of Aldrin in DU145 PCa cells. To this aim, two complementary approaches combining model-driven and data-driven techniques were applied. This strategy facilitated the investigation of metabolic dysregulations from a mechanistic point of view by means of constraint-based modeling techniques while finding potential gene regulatory or signaling mechanisms by applying GSEA.

Finally, a comprehensive literature review was conducted to investigate the cross-talk between metabolic and gene regulatory processes and establish a single mechanism explaining the increased likelihood of developing malignancy in cases of a chronic exposure to Aldrin in PCa. Experimental validations confirmed these findings, while extensive support from the literature further reinforced them.

Our multi-level approach has enabled us to identify various alterations in important metabolic pathways. Specifically, changes were detected in eicosanoids, glycerophospholipids, folate, N-glycan synthesis, NAD, as well as key amino acids such as valine, leucine, isoleucine, thiamine, and glutathione metabolism. Thus, tumorigenic activity has been reported in some eicosanoid metabolism intermediates [44]; it is also reported that glycerophospholipids [45] and folate [46] are positively correlated with an increased risk of aggressive prostate cancer. An N-glycomic profile has been described as a biomarker in PCa, which highlights the relevance of this pathway in PCa progression and malignancy acquisition [47]. Several studies have established a link between the amino acids described in our research and their involvement in the progression of PCa and the acquisition of malignancy [48,49,50]. In addition, a key role of glutathione has been associated with ferroptosis, a recently described apoptotic mechanism that is altered in PCa [51] (Figure 2a). Furthermore, a unified cross-talk mechanism involving the metabolism and gene regulation associated with the chronic exposure to Aldrin in DU145 cells was identified. Our findings are consistent with the up-regulation of PLIN2 and INSIG2 that enhance arachidonoyl-CoA degradation via beta oxidation and cholesterol metabolism inhibition via HMGCoA ubiquitination, respectively (Figure 2e). Therefore, the ratio of Cholesterol/TAG was modified due to the competition between these two processes for the Acetil-CoA pool. This observation has been reported in DU-145 cells following a prolonged exposure to non-lethal levels of Aldrin [6].

Furthermore, we employed PLS-DA analysis to identify the specific set of genes associated with a chronic exposure to Aldrin. We then utilized GSEA to compare this set of genes with the TGCA database. We discovered a strong correlation between the genes modified in DU-145 prostate cancer cells following a chronic exposure to Aldrin and tumor progression and metastasis in up to eight distinct cancer types. This implies that this approach has the potential to be utilized in other cancer types.

In this study, we have identified a potential cellular mechanism underlying the gain of malignancy associated with the chronic exposure to Aldrin in PCa cells. This approach allows the study of different levels in the cell hierarchy, such as genomic, proteomic, or metabolomic, as well as the cross-talk between them. Here, the gene dysregulation discovered through gene set enrichment analysis (GSEA) has a significant influence on the levels of HMGCoA protein. This, in turn, directly impacts the metabolism and affects the ratio TAG/Cholesterol as well as the gene regulatory levels. These findings align with the alterations observed in the GEM analysis and are consistent with the results reported by Bedia et al. [6].

## 5. Conclusions

Metabolic reactions require enzymatic catalysis to achieve the appropriate rate of activity due to their proximity to thermodynamic equilibrium. Enzymatic levels and activity are tightly regulated through intricate gene regulatory pathways and metabolic processes. This underscores the crucial involvement of proteins in regulating the metabolism and their contribution to diseases characterized by dysregulated metabolic functions.

Factors that affect enzyme activity include the reactants, substrate, inhibitors, pH, temperature, and different proteoforms [52]. The role of proteoforms and their production by alternative splicing, sequence variations, and post-translational modifications, among others, have been highlighted in prostate cancer [53].

In this work, we have studied different levels of the cell hierarchical mechanisms by applying two different and complementary approaches, CBM and GSEA, to analyze gene regulation and metabolism using multiple omics data, including transcriptomic, metabolomic, lipidomic, and literature-based data.

Our computational analysis revealed that the dysregulation of the HMGCoA enzyme by the SOX4 gene through INSIG2 in cells exposed to Aldrin altered the ratio of TAG/cholesterol, leading to an enhanced malignant phenotype in these cells. This method provides a holistic perspective that enables a more complete understanding of cellular processes.

Furthermore, we also established a correlation between the observed changes in the gene expression profile of Aldrin in DU145 cells after a chronic exposure to Aldrin and the progression of the tumor in different cancer types, providing crucial insights into the underlying mechanisms governing cancer onset and development.

In the present work, two main goals have been achieved. First, we developed a comprehensive computational pipeline to investigate the hierarchical molecular mechanisms occurring within a cell’s various layers. This computational approach presents a significant conceptual shift in the way we study the metabolism and gene regulation. Rather than viewing the metabolism as the final product of gene regulation, this study proposes a bi-directional cross-talk between the metabolic, proteomic, and gene regulatory levels, where changes in either of them can affect the others. Second, a unified mechanism underlying the enhanced malignancy in PCa following a chronic exposure to Aldrin has been described.

The approach presented here offers a comprehensive tool for studying the molecular mechanisms that drive metabolic reprogramming in response to a chronic exposure to sub-lethal concentrations of pollutants. This not only allows for a more holistic understanding of the underlying processes but deepens our knowledge of the intricate interplay between these systems and opens up new avenues for research and discovery, with potential clinical and environmental applications.

## Figures and Tables

**Table 1:** List of overlapped genes between MENSE_HYPOXIA_UP and PRAMOONJAGO_SOX4_TARGETS_UP gene sets.

Gene ID	Gene Name	Ensembl ID
MXI1	MAX interactor 1, dimerization protein	ENSG00000119950
EGLN1	egl-9 family hypoxia inducible factor 1	ENSG00000135766
PEX13	Peroxisomal biogenesis factor 13	ENSG00000162928
MAFF	MAF bZIP transcription factor F	ENSG00000185022
RNASE4	Ribonuclease A family member 4	NSG00000258818
PPFIA4	PTPRF interacting protein alpha 4	ENSG00000143847
PPP1R3C	Protein phosphatase 1 regulatory subunit 3C	ENSG00000119938
C7orf68	Hypoxia inducible lipid droplet-associated	ENSG00000135245
GPI	Glucose-6-phosphate isomerase	ENSG00000105220
PLIN2	Perilipin 2	ENSG00000147872
RIOK3	RIO kinase 3	ENSG00000101782
NDRG1	N-myc downstream regulated 1	ENSG00000104419
INSIG2	Insulin-induced gene 2	ENSG00000125629
NFIL3	Nuclear factor, interleukin 3 regulated	ENSG00000165030

**Table 2:** Tumor types from the TCGA with progression and metastasis that correlate with the gene set associated with the gain of malignancy in DU145 PCa cells due to the chronic exposure to Aldrin. ES: enrichment score, NES: normalized enrichment score, FDR: false discovery rate, No. samples: number of samples.

Cancer ID	Cancer Name	ES	NES	FDR	No. Samples
LUAD	Lung adenocarcinoma	0.41	1.64	0.01	488
BLCA	Bladder urothelial carcinoma	0.37	1.48	0.043	128
KIRP	Kidney renal papillary carcinoma	0.37	1.47	0.05	243
READ	Rectum adenocarcinoma	0.36	1.41	0.052	93
UVM	Uveal melanoma	0.33	1.34	124	27
DBLC	Lymphoid neoplasm diffuse large B-cell lymphoma	0.35	1.34	0.151	35
KICH	Kidney chromophobe	0.31	1.24	0.187	66
CESC	Cervical squamous cell carcinoma and endocervical adenocarcinoma	0.32	1.2	0.2	211

## Data Availability

All data generated or analyzed in this study are either included in the main text and Supplementary Materials, or the transcriptomic data are deposited in GEO database (GSE132063—https://www.ncbi.nlm.nih.gov/geo/query/acc.cgi?acc=GSE132063, accessed on 15 December 2022).

## Supplementary Materials

The following supporting information can be downloaded at: https://www.mdpi.com/article/10.3390/proteomes11020011/s1, Supplementary Material S1: Experimental data pre-treatment; Supplementary Material S2: Data-driven analysis (GSEA); Supplementary Material S3: Model-driven analysis (CBM); Supplementary Material S4: Metabolic modeling validation: cell viability, Western blot and cholesterol measurements; Supplementary Material S5: GSEA determining correlation between relevant genes associated with the chronic exposure to Aldrin in DU145 cells and tumors retrieved from TGCA.
